# 肿瘤溶解综合征对儿童成熟B细胞淋巴瘤预后的影响

**DOI:** 10.3760/cma.j.cn121090-20240624-00234-1

**Published:** 2024-12

**Authors:** 成功 曾, 芷晴 韦, 俊廷 黄, 佳 朱, 斐斐 孙, 娟 王, 素英 路, 翼鷟 张, 晓非 孙, 子俊 甄

**Affiliations:** 中山大学肿瘤防治中心儿童肿瘤科，华南恶性肿瘤防治全国重点实验室，广东省鼻咽癌诊治研究重点实验室，广东省恶性肿瘤临床医学研究中心，广州 510060 Department of Pediatric Oncology, State Key Laboratory of Oncology in South China, Guangdong Provincial Clinical Research Center for Cancer, Guangdong Key Laboratory of Nasopharyngeal Carcinoma Diagnosis and Therapy, Sun Yat-sen University Cancer Center, Guangzhou 510060, China

**Keywords:** B细胞淋巴瘤, 儿童, 青少年, 肿瘤溶解综合征, 预后, B-cell lymphoma, Child, Adolescent, Tumor lysis syndrome, Prognosis

## Abstract

**目的:**

探讨肿瘤溶解综合征（TLS）对中高风险组儿童青少年高级别成熟B细胞非霍奇金淋巴瘤（HG B-NHL）患者预后的影响。

**方法:**

收集2010年1月至2022年12月中山大学肿瘤防治中心儿童肿瘤科收治的18岁以下的初治中高风险组HG B-NHL共283例患者的临床资料及预后情况。对患者的临床特征、发生TLS时的实验室指标及预后进行分析，实验室指标的最佳截断值使用R软件根据无事件生存（EFS）进行确定。

**结果:**

中位年龄7（1～18）岁，男女比例为3.6∶1。76例（26.9％）发生TLS，207例（73.1％）未发生TLS。与无TLS的患者相比，TLS患者的病理亚型为伯基特淋巴瘤、危险分层为高风险、年龄<12岁、LDH≥1 000 IU/L的比例均更高（均*P*<0.05）。全组患者的5年EFS率及总生存（OS）率分别为（84.5±2.2）％和（88.2±2.0）％。发生TLS患者的5年OS率低于未发生TLS的患者［（80.8±4.6）％对（91.0±2.0）％，*P*＝0.01）］。发生TLS时，与血尿酸>612.7 µmol/L的患者（40例）相比，血尿酸≤612.7 µmol/L的患者（36例）5年EFS率［（67.8±8.1）％对（87.5±5.2）％，*P*＝0.04］及OS率［（69.9±8.1）％对（90.0±4.7）％，*P*＝0.04］较低；与血磷>1.89 mmol/L的患者（18例）相比，血磷≤1.89 mmol/L的患者（58例）5年EFS率［（71.6±6.0）％对100％，*P*＝0.02］及OS率［（74.8±5.8）％对100％，*P*＝0.03］较低。

**结论:**

TLS与HG B-NHL患者的不良预后相关。发生TLS时血尿酸和血磷水平较低的患者预后更差，对预测患者预后及指导分层治疗具有一定价值。

## Abstract (en)

**Objective:**

This study aimed to investigate the effect of tumor lysis syndrome（TLS）on the prognosis of children and adolescents with intermediate- or high-risk high-grade mature B-cell nonHodgkin lymphoma（HG B-NHL）.

**Methods:**

This study collected the clinical data and prognosis of 283 patients aged <18 years with newly diagnosed intermediate- or high-risk HG B-NHL treated at the Sun Yat-sen University Cancer Center from January 2010 to December 2022. The clinical characteristics, laboratory indicators during TLS, and prognosis of the patients were analyzed. The optimal cutoff values of laboratory indicators during TLS were identified using R studio according to event-free survival（EFS）.

**Results:**

Of the 283 patients enrolled, the median age was 7（range: 1–18）years and the male-to-female ratio was 3.6∶1, 76（26.9％）developed TLS, and 207（73.1％）did not. Patients with TLS demonstrated higher proportions of the pathological subtype Burkitt lymphoma, high-risk stratification, age <12 years, and LDH of ≥1 000 IU/L compared with patients without TLS（all *P*<0.05）. The 5-year EFS and overall survival（OS）rates of the entire group were（84.5±2.2）％ and（88.2±2.0）％, respectively. The 5-year OS rate of patients with TLS was significantly lower than that of those without TLS［（80.8±4.6）％ *vs*（91.0±2.0）％, *P*＝0.01］. Among patients with TLS, those with serum uric acid of ≤612.7 µmol/L（*n*＝36）exhibited lower 5-year EFS［（67.8±8.1）％ *vs*（87.5±5.2）％, *P*＝0.04］and OS rates［（69.9±8.1）％ *vs*（90.0±4.7）％, *P*＝0.04］compared with those with uric acid of >612.7 µmol/L（*n*＝40）. Similarly, patients with serum phosphate of ≤1.89 mmol/L（*n*＝58）demonstrated lower 5-year EFS［（71.6±6.0）％ *vs* 100％, *P*＝0.02］and OS rates［（74.8±5.8）％ *vs* 100％, *P*＝0.03］compared with those with phosphate of >1.89 mmol/L（*n*＝18）.

**Conclusion:**

TLS is associated with poor prognosis in patients with HG B-NHL. Patients with lower serum uric acid and phosphate levels during TLS demonstrated worse prognoses, indicating their potential value in predicting prognosis and guiding stratified treatment.

B细胞非霍奇金淋巴瘤（B-NHL）是儿童青少年最常见的非霍奇金淋巴瘤（NHL）亚型，占所有NHL的60％[1]。在儿童B-NHL中，80％为伯基特淋巴瘤（BL），10％～20％为弥漫大B细胞淋巴瘤（DLBCL）[2]。BL、DLBCL和高级别B细胞淋巴瘤非特指型（HGBCL，NOS）在病理上被归类为高级别成熟B细胞非霍奇金淋巴瘤（HG B-NHL）[3]–[4]。

儿童青少年HG B-NHL患者接受法国-美国-英国/恶性B淋巴瘤（FAB/LMB）方案或柏林-法兰克福-蒙斯特（BFM）方案治疗，5年总生存（OS）率可达90％以上[5]–[7]。然而仍然有约20％的HG B-NHL患者会出现复发或进展。复发或进展的患者即使接受了挽救化疗或造血干细胞移植，5年OS率仍<30％[8]–[10]。因此，提前识别出这部分患者对于进一步提高儿童青少年HG B-NHL患者的治疗效果具有重要意义。目前，用于HG B-NHL危险分层的预后因素不多。BFM方案根据肿瘤分期、部位、手术切除程度以及血清LDH水平等将HG B-NHL分为4个风险组（R1～R4）。FAB/LMB方案则是根据肿瘤分期和手术切除程度将患者分为3个风险组（A、B、C）。开发更多预后因素有助于提高治疗的个体化程度。

肿瘤溶解综合征（TLS）是由肿瘤细胞的快速、大量破坏引起的肿瘤急症，最常见于化疗开始后高度增殖的恶性肿瘤，如急性白血病和侵袭性淋巴瘤[11]。晚期HG B-NHL患者TLS的发生率可达20％～30％，25％的患者在诱导化疗期间需要血液透析支持[12]–[13]。TLS可分为实验室TLS（LTLS）和临床TLS（CTLS），CTLS患者的死亡风险更大[14]–[15]。研究表明TLS对多发性骨髓瘤及HIV相关NHL患者的预后有不良影响[16]–[17]。然而，目前关于TLS对儿童青少年HG B-NHL预后影响的研究不多。本研究回顾了本中心治疗的中高风险组儿童青少年HG B-NHL患者，分析了TLS对中高风险组HG B-NHL患者预后的影响。

## 病例与方法

1. 病例：2010年1月至2022年12月期间，在中山大学肿瘤防治中心治疗的18岁以下的初诊中高风险组HG B-NHL患者共283例入组。研究获得中山大学肿瘤防治中心伦理审查委员会批准（批件号：B2023-728-01），所有患者均签署知情同意书。

2. 诊断与分期：所有患者经病理诊断为BL、DLBCL或HGBCL，NOS。肿瘤分期参照国际儿童NHL分期系统[18]。骨髓侵犯定义为骨髓中肿瘤细胞>5％。符合以下任何一项被定义为中枢神经系统侵犯：脑脊液中发现幼稚淋巴瘤细胞；孤立的脑内肿块；颅神经麻痹；脊柱旁或脑膜旁病灶延伸；病灶扩散至脊髓或颅内。

3. 危险分层：参照BFM方案危险分层，根据患者肿瘤分期、手术切除程度、LDH水平、中枢神经系统、骨髓及脑膜旁侵犯将HG B-NHL分为极低风险（R1）、低风险（R2）、中风险（R3）和高风险（R4）4个风险组[19]，脑膜旁被定位为中耳、乳突、鼻咽、鼻窦、咽旁间隙、颞下窝/翼腭窝。

4. 治疗方法：所有患者按我中心的改良BFM方案化疗，具体用药及剂量见文献[19]。根据不同危险分层，采用不同强度方案交替化疗。ANC和PLT经过最低点后分别回升至0.5×10^9^/L和100×10^9^/L以上后开始下一个疗程的化疗，最长化疗间隔时间不超过3周。R1组接受A（地塞米松+异环磷酰胺+长春新碱+甲氨蝶呤+阿糖胞苷+依托泊苷）方案和B（地塞米松+长春新碱+环磷酰胺+甲氨蝶呤+阿霉素）方案交替2个疗程。R2组接受V（泼尼松+环磷酰胺）方案减瘤后，A和B方案交替4个疗程。R3组接受V方案减瘤后，R-AA（利妥昔单抗+地塞米松+异环磷酰胺+长春新碱+甲氨蝶呤+阿糖胞苷+依托泊苷）方案、R-BB（利妥昔单抗+地塞米松+长春新碱+环磷酰胺+甲氨蝶呤+阿霉素）方案和R-CC（利妥昔单抗+地塞米松+长春地辛+阿糖胞苷+依托泊苷）方案交替5个疗程。R4组接受V方案减瘤后，R-AA、R-BB和R-CC方案交替6个疗程。所有患者每个疗程接受腰椎穿刺及鞘内注射化疗药物。

5. TLS诊断标准：采用Cairo-Bishop诊断标准定义TLS[11],[20]。LTLS定义为减瘤化疗V方案开始前3 d至开始后7 d内出现以下2项及以上实验室指标异常：血尿酸≥476 µmol/L或较基线升高25％以上；血钾≥6.0 mmol/L或较基线升高25％以上；血磷≥2.1 mmol/L或较基线升高25％以上；血钙≤1.75 mmol/L或较基线降低25％以上。本研究采用的发生TLS时实验室指标为诊断TLS时的数据。CTLS定义为LTLS加以下任意1项及以上：血肌酐≥1.5倍正常值上限；心律失常；急性发作的抽搐；猝死。患者诊断TLS后，应用水化、利尿剂、拉布立酶治疗，必要时转入重症监护病房血液透析治疗。

6. 随访及研究终点：采用门诊复查及电话随访，末次随访日期为2024年1月31日。患者结束治疗后的2年内每3个月复查1次，3～5年每半年复查1次，5年及以上每年复查1次。主要研究终点为无事件生存（EFS）和OS。EFS期定义为自诊断为HG B-NHL起至第一次疾病进展、疾病复发、任何原因导致的死亡或第二恶性肿瘤发生的时间。OS期定义为自诊断为HG B-NHL起至任何原因导致的死亡时间。

7. 统计学处理：计数资料以例数（百分比）表示，计量资料以*M*（范围）或*x*±*s*表示。OS及EFS采用Kaplan-Meier的方法进行估算，采用Log-rank检验比较组间不良事件的风险。采用Cox风险回归模型对影响EFS和OS的危险因素进行单因素及多因素分析。用卡方检验比较各亚组临床特征的差异。发生TLS时血尿酸、血钾、血磷、血钙的最佳截断值采用R studio version 4.3.1根据EFS进行确定。采用*t*检验及Mann-Whitney检验比较各指标在不同亚组间分布差异。统计软件采用SPSS Statistics 25及GraphPad Prism 10.0软件，所有的检验均为双侧，*P*<0.05为差异具有统计学意义。

## 结果

1. 临床特征：见表1，283例患者中，发生TLS 76例（26.9％），未发生TLS 207例（73.1％）。中位年龄7（1～18）岁，男女比例为3.6:1。诊断时LDH中位值为705 IU/L。其中Ⅲ期、Ⅳ期患者分别有196例（69.3％）和87例（30.7％）。危险分层为R3的患者有141例（49.8％）、R4有142例（50.2％）。与无TLS的患者相比，TLS患者的病理亚型为BL、危险分层为R4、年龄<12岁、LDH≥1 000 IU/L的比例均更高（均*P*<0.05）。

**表1:** 有无发生TLS的儿童青少年高级别成熟B细胞非霍奇金淋巴瘤患者的临床特征比较［例（％）］

临床特征	未发生TLS（207例）	发生TLS（76例）	*χ*^2^值	*P*值
性别			0.73	0.42
男	165（79.7）	57（75.0）		
女	42（20.3）	19（25.0）		
年龄			6.94	0.01
<12岁	159（76.8）	69（90.8）		
≥12岁	48（23.2）	7（9.2）		
病理类型			12.85	<0.001
BL	157（75.8）	72（94.7）		
DLBCL/HGBCL，NOS	50（24.2）	4（5.3）		
肿瘤分期			0.03	0.88
Ⅲ期	144（69.6）	52（68.4）		
Ⅳ期	63（30.4）	24（31.6）		
危险分层			48.14	<0.001
R3	129（62.3）	12（15.8）		
R4	78（37.7）	64（84.2）		
骨髓			2.57	0.14
肿瘤侵犯	39（18.8）	21（27.6）		
无肿瘤侵犯	168（81.2）	55（72.4）		
中枢神经系统			3.86	0.07
肿瘤侵犯	32（15.5）	5（6.6）		
无肿瘤侵犯	175（84.5）	71（93.4）		
LDH			81.36	<0.001
<1 000 IU/L	160（77.3）	14（18.4）		
≥1 000 IU/L	47（22.7）	62（81.6）		

**注** TLS：肿瘤溶解综合征；BL：伯基特淋巴瘤；DLBCL：弥漫大B细胞淋巴瘤；HGBCL，NOS：高级别B细胞淋巴瘤，非特指型；R3：中风险；R4：高风险

2. TLS诊断与分型：发生LTLS的患者有57例（20.1％），发生CTLS的患者有19例（6.7％）。在TLS患者中，血尿酸升高76例（100％）、血钾升高27例（35.5％）、血磷升高37例（48.7％）、血钙降低27例（35.5％）、心律失常11例（14.5％）、急性抽搐7例（9.2％）、猝死0例，具体见表2。发生TLS时血尿酸、血钾、血磷、血钙中位浓度分别为643.5（383.0～1 387.0）µmol/L、4.39（2.87～6.68）mmol/L、1.62（0.79～4.31）mmol/L和2.17（1.30～2.81）mmol/L。进一步分析发现TLS患者中，血尿酸在男性患者中较低（*P*＝0.04），血钾在年龄较小［即<中位年龄（6.9岁）］的患者中较高（*P*＝0.01），发生TLS时血尿酸、血钾、血磷和血钙在其余临床特征（病理亚型、肿瘤分期、危险分层、骨髓侵犯、中枢神经系统侵犯、LDH水平）中的分布差异均无统计学意义（均*P*>0.05）。

**表2:** 儿童青少年高级别成熟B细胞非霍奇金淋巴瘤患者诊断肿瘤溶解综合征（TLS）时的实验室和临床指标

诊断TLS的相关指标	例数（％）
血尿酸	
≥476 µmol/L	66（86.8）
<476 µmol/L但较基线升高25％以上	10（13.2）
<476 µmol/L且较基线升高小于25％	0（0）
血钾	
≥6.0 mmol/L	7（9.2）
<6.0 mmol/L但较基线升高25％以上	20（26.3）
<6.0 mmol/L且较基线升高小于25％	49（64.5）
血磷	
≥2.1 mmol/L	6（7.9）
<2.1 mmol/L但较基线升高25％以上	31（40.8）
<2.1 mmol/L且较基线升高小于25％	39（51.3）
血钙	
≤1.75 mmol/L	10（13.2）
>1.75 mmol/L但较基线降低25％以上	17（22.4）
>1.75 mmol/L且较基线降低小于25％	49（64.4）
血肌酐	
≥1.5倍的正常值上限	10（13.2）
<1.5倍的正常值上限	66（86.8）

3. 生存分析：中位随访时间为61.9（1.4～171.1）个月。全组患者的5年EFS率及OS率分别为（84.5±2.2）％和（88.2±2.0）％。R3组和R4组患者的5年EFS率分别为（87.7±2.8）％和（81.3±3.3）％（*P*＝0.15，图1A），5年OS率分别为（92.4±2.3）％和（84.0±3.1）％（*P*＝0.03，图1B）。Ⅲ期和Ⅳ期患者的5年EFS率分别为（85.9±2.5）％和（81.5±4.2）％（*P*＝0.36），5年OS率分别为（89.8±2.2）％和（84.8±3.9）％（*P*＝0.23）。

**图1:**
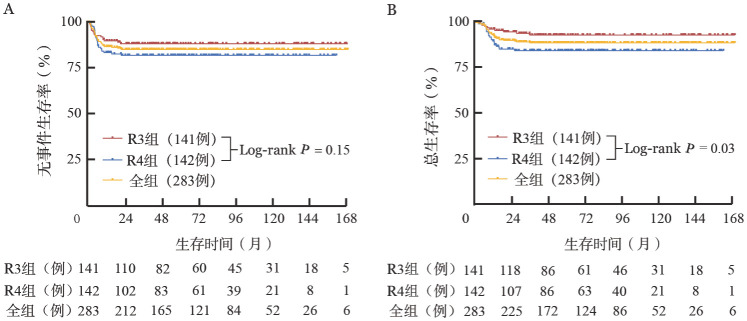
不同危险分层的高级别成熟B细胞非霍奇金淋巴瘤患者的无事件生存（A）和总生存（B）曲线

发生TLS（76例）和未发生TLS（207例）患者的5年EFS率分别为（78.4±4.8）％和（86.8±2.4）％（*P*＝0.10，图2A），5年OS率分别为（80.8±4.6）％和（91.0±2.0）％（*P*＝0.01，图2B）。LTLS和CTLS患者的5年EFS率分别为（80.0±5.4）％和（73.7±10.1）％（*P*＝0.50），5年OS率分别为（83.1±5.2）％和（73.7±10.1）％（*P*＝0.27）。发生CTLS的患者5年OS率低于未发生TLS的患者（*P*<0.01）。

**图2:**
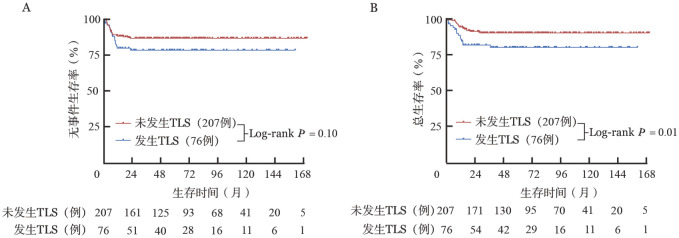
是否发生肿瘤溶解综合征（TLS）的高级别成熟B细胞非霍奇金淋巴瘤患者的无事件生存（A）和总生存（B）曲线

根据患者的EFS，发生TLS时的血尿酸、血钾、血磷和血钙的最佳截断值分别为612.7 µmol/L、5.17 mmol/L、1.89 mmol/L和2.08 mmol/L。发生TLS时血尿酸≤612.7 µmol/L的患者（36例）5年EFS率［（67.8±8.1）％对（87.5±5.2）％，*P*＝0.04，图3A］及OS率［（69.9±8.1）％对（90.0±4.7）％，*P*＝0.04，图3B］低于血尿酸>612.7 µmol/L的患者（40例）。发生TLS时血磷≤1.89 mmol/L的患者（58例）5年EFS率［（71.6±6.0）％对100％，*P*＝0.02，图3C］及OS率［（74.8±5.8）％对100％，*P*＝0.03，图3D］低于血磷>1.89 mmol/L的患者（18例）。由于发生CTLS的患者预后显著较差，进一步根据患者的EFS，得到发生CTLS时血尿酸、血钾、血磷和血钙的最佳截断值分别为735.9 µmol/L、3.20 mmol/L、1.27 mmol/L和2.14 mmol/L。发生CTLS时血磷≤1.27 mmol/L的患者（4例）5年EFS率［（25.0±21.7）％对（86.7±8.8）％，*P*＝0.02］及OS率［（25.0±21.7）％对（86.7±8.8）％，*P*＝0.02］低于血磷>1.27 mmol/L的患者（15例）。其余指标（肿瘤分期、发生TLS时血钙、发生CTLS时血尿酸、发生CTLS时血钾、发生CTLS时血钙）的生存率差异均无统计学意义（均*P*>0.05）。

**图3:**
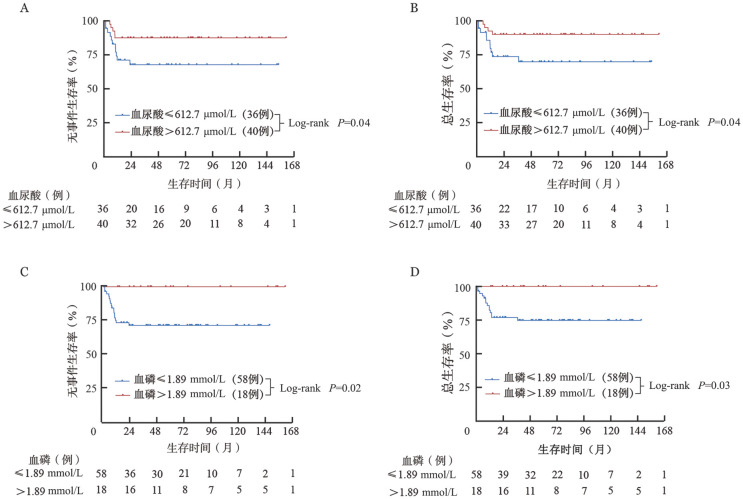
根据血尿酸及血磷分层的肿瘤溶解综合征患者的生存曲线 **A** 按血尿酸分层的无事件生存曲线；**B** 按血尿酸分层的总生存曲线； **C** 按血磷分层的无事件生存曲线；**D** 按血磷分层的总生存曲线

5. 单因素及多因素分析：如表3所示，在单因素分析中，病理类型为BL［*HR*＝3.24（95％ *CI* 1.52～6.88），*P*＝0.04］、血清LDH≥1 000 IU/L［*HR*＝0.41（95％ *CI* 0.22～0.75），*P*<0.01］的患者5年EFS率较差，病理类型为BL［*HR*＝7.49（95％ *CI* 3.12～17.96），*P*＝0.02］、血清LDH≥1 000 IU/L［*HR*＝0.24（95％ *CI* 0.12～0.48），*P*<0.001］、发生TLS［*HR*＝0.43（95％ *CI* 0.19～0.96），*P*＝0.01］的患者5年OS率较差。在多因素Cox分析中，血清LDH≥1 000 IU/L为患者EFS［*HR*＝2.10（95％ *CI* 1.12～3.95），*P*＝0.02］和OS［*HR*＝3.33（95％ *CI* 1.40～7.94），*P*<0.01］的独立不良预后因素。

**表3:** 儿童青少年HG B-NHL患者的单因素和多因素分析

变量	无事件生存				总生存			
	单因素分析		多因素分析		单因素分析		多因素分析	
	*HR*（95％ *CI*）	*P*值	*HR*（95％ *CI*）	*P*值	*HR*（95％ *CI*）	*P*值	*HR*（95％ *CI*）	*P*值
性别								
男	2.10（1.02~4.35）	0.12		N/A	1.46（0.62~3.40）	0.44		N/A
女	0.48（0.23~0.98）				0.69（0.29~1.60）			
年龄								
<6.9岁	1.17（0.64~2.12）	0.62		N/A	1.12（0.56~2.24）	0.75		N/A
≥6.9岁	0.86（0.47~1.56）				0.89（0.45~1.79）			
病理类型								
BL	3.24（1.52~6.88）	0.04	2.37（0.71~7.94）	0.16	7.49（3.12~17.96）	0.02	4.32（0.57~32.88）	0.16
DLBCL/HGBCL，NOS	0.31（0.15~0.66）				0.13（0.06~0.32）			
分期								
Ⅲ期	0.75（0.39~1.43）	0.35		N/A	0.65（0.31~1.38）	0.23		N/A
Ⅳ期	1.34（0.70~2.56）				1.54（0.73~3.26）			
骨髓								
无肿瘤侵犯	0.79（0.38~1.63）	0.49		N/A	0.82（0.35~1.90）	0.62		N/A
肿瘤侵犯	1.27（0.61~2.65）				1.23（0.53~2.86）			
中枢神经系统								
无肿瘤侵犯	0.64（0.26~1.57）	0.25		N/A	0.65（0.23~1.83）	0.34		N/A
肿瘤侵犯	1.57（0.64~3.84）				1.53（0.55~4.27）			
LDH								
<1 000 IU/L	0.41（0.22~0.75）	<0.01			0.24（0.12~0.48）	<0.001		
≥1 000 IU/L	2.45（1.33~4.54）		2.10（1.12~3.95）	0.02	4.25（2.08~8.69）		3.33（1.40~7.94）	<0.01
TLS								
未发生	0.60（0.30~1.18）	0.10		N/A	0.43（0.19~0.96）	0.01		
发生	1.67（0.85~3.29）				2.32（1.05~5.17）		1.07（0.49~2.34）	0.86

**注** HG B-NHL：高级别成熟B细胞非霍奇金淋巴瘤；TLS：肿瘤溶解综合征；BL：伯基特淋巴瘤；DLBCL：弥漫大B细胞淋巴瘤；HGBCL，NOS：高级别B细胞淋巴瘤，非特指型；N/A：未计算

## 讨论

TLS多发生于肿瘤负荷较大的具有高度侵袭性的淋巴造血系统肿瘤，在本研究的R3/R4组儿童青少年HG B-NHL患者中的发生率为26.9％，而且较多发生于病理亚型为BL、R4组和化疗前血清LDH≥1 000 IU/L的患者。LDH升高及肿瘤分期较晚是恶性肿瘤患者TLS发生的重要危险因素，而BL则被认为是最容易发生TLS的肿瘤类型之一[21]，本研究再次证实了这一观点。

本研究进一步发现，发生TLS的患者5年OS率显著低于未发生TLS的患者（80.8％对91.0％）。TLS可以激发和促进细胞因子释放综合征（CRS）的发生[16],[22]。CRS释放的大量细胞因子如IL-6、γ干扰素和TNF-α，通过影响肿瘤微环境，从而促进肿瘤的扩散和转移[23]。这些细胞因子与淋巴瘤患者的高肿瘤负荷、低治疗反应率和较差的预后相关[24]–[25]。这可能是TLS导致HG B-NHL患者预后较差的潜在机制。此外，本研究中TLS在R4组和LDH≥1 000 IU/L的患者中发生比例较高，提示多种因素导致患者预后差。

CTLS是在满足LTLS的诊断标准的基础上，对患者的脏器造成了损害。本研究中，CTLS在R3/R4组儿童青少年HG B-NHL患者中的发生率为6.7％。伴有CTLS的患者5年EFS率及OS率较差，均为73.7％。研究发现急性肾损伤是导致TLS患者死亡的主要原因，住院期间死亡比值比高达10.41[26]–[28]。因此，TLS导致的危及生命的脏器损害也是导致TLS患者预后较差的原因之一。

肿瘤细胞的快速和大量破坏引起的细胞内物质释放到体循环中是TLS发生的本质，血尿酸升高是TLS发生的一个标志性事件，发生于本研究中所有TLS患者。恶性肿瘤细胞中的磷含量可以达到正常细胞中磷含量的4倍，这些磷的快速释放也会导致高磷血症[29]，在本研究中发生率达48.7％。在发生TLS的患者中，与血尿酸或血磷水平较低的患者相比，血尿酸>612.7 µmol/L或血磷>1.89 mmol/L的患者预后较好。一方面，可能是由于较高的血尿酸和血磷水平提示肿瘤细胞对化疗药物敏感度高，短期内肿瘤细胞被大量破坏[30]。治疗主要依靠化疗，高化疗敏感性导致更好的预后。另一方面，在减瘤化疗阶段血磷和血尿酸水平较高的患者通常能更及时地被发现发生TLS，能更快的转至重症监护室接受血液透析治疗，尽快减少各种细胞因子及代谢产物对患者器官的损伤以及对肿瘤的促进作用。此外，血尿酸和血磷水平在R4组和LDH≥1 000 IU/L的患者中分布差异无统计学意义，提示其预后意义并不受其他预后因素干扰。

血尿酸和血磷是发生TLS时过多积聚的代谢产物，本研究首次在儿童青少年HG B-NHL并发TLS的患者中证实这些代谢产物水平越高，预后越好，值得进一步探讨。但研究有一定局限性。本文为回顾性分析，不排除有研究偏倚，样本量较小，统计效力差，需要更大的样本量进一步验证。

综上所述，发生TLS的患者与较高的危险分层及较高的LDH水平相关，预后较差。而发生TLS时血尿酸和血磷水平较高的患者预后较好。因此，TLS及发生TLS时的血尿酸和血磷水平未来可作为预测患者预后、指导临床治疗的预测因素。
